# Performance of a capnodynamic method estimating effective pulmonary blood flow during transient and sustained hypercapnia

**DOI:** 10.1007/s10877-017-0021-3

**Published:** 2017-05-11

**Authors:** Thorir Svavar Sigmundsson, Tomas Öhman, Magnus Hallbäck, Eider Redondo, Fernando Suarez Sipmann, Mats Wallin, Anders Oldner, Caroline Hällsjö Sander, Håkan Björne

**Affiliations:** 1 Department of Perioperative Medicine and Intensive Care, Karolinska University Hospital, Stockholm, Sweden; 2 Department of Physiology and Pharmacology, Karolinska Institutet, Stockholm, Sweden; 3 Maquet Critical Care AB, Solna, Sweden; 4 Department of Intensive Care Medicine, Hospital de Navarra, Pamplona, Spain; 5 Hedenstierna’s laboratory, Section of Anaesthesiology and Critical Care, Department of Surgical Sciences, Uppsala University, Uppsala, Sweden; 6 CIBER de Enfermedades Respiratorias, Instituto de Salud Carlos III, Madrid, Spain

**Keywords:** Carbon dioxide, Cardiac output, Intraoperative monitoring, Effective pulmonary blood flow, Capnodynamic, Animal model

## Abstract

The capnodynamic method is a minimally invasive method continuously calculating effective pulmonary blood flow (CO_EPBF_), equivalent to cardiac output when intra pulmonary shunt flow is low. The capnodynamic equation joined with a ventilator pattern containing cyclic reoccurring expiratory holds, provides breath to breath hemodynamic monitoring in the anesthetized patient. Its performance however, might be affected by changes in the mixed venous content of carbon dioxide (C_v_CO_2_). The aim of the current study was to evaluate CO_EPBF_ during rapid measurable changes in mixed venous carbon dioxide partial pressure (P_v_CO_2_) following ischemia–reperfusion and during sustained hypercapnia in a porcine model. Sixteen pigs were submitted to either ischemia–reperfusion (n = 8) after the release of an aortic balloon inflated during 30 min or to prolonged hypercapnia (n = 8) induced by adding an instrumental dead space. Reference cardiac output (CO) was measured by an ultrasonic flow probe placed around the pulmonary artery trunk (CO_TS_). Hemodynamic measurements were obtained at baseline, end of ischemia and during the first 5 min of reperfusion as well as during prolonged hypercapnia at high and low CO states. Ischemia–reperfusion resulted in large changes in P_v_CO_2_, hemodynamics and lactate. Bias (limits of agreement) was 0.7 (−0.4 to 1.8) L/min with a mean error of 28% at baseline. CO_EPBF_ was impaired during reperfusion but agreement was restored within 5 min. During prolonged hypercapnia, agreement remained good during changes in CO. The mean polar angle was −4.19° (−8.8° to 0.42°). Capnodynamic CO_EPBF_ is affected but recovers rapidly after transient large changes in P_v_CO_2_ and preserves good agreement and trending ability during states of prolonged hypercapnia at different levels of CO.

**Electronic supplementary material:**

The online version of this article (doi:10.1007/s10877-017-0021-3) contains supplementary material, which is available to authorized users.

## Introduction

Hemodynamic optimisation in high-risk surgery patients using goal directed protocols has shown to improve postoperative outcomes and is assumed to be cost effective [1–5]. A flow based parameter like cardiac output (CO) is ideally used to guide hemodynamic support [3]. Patients accepted for surgery nowadays tend to be older and carry more co-morbidity, thus expanding the high-risk group [6]. This underlines the need for a feasible CO monitor which is both fast and accurate during hemodynamic changes [7]. Several new methods for CO monitoring have emerged in recent years but have different limitations and most have not been adequately evaluated during hemodynamic alterations [8, 9].

Alveolar carbon dioxide concentration in expired gas can be easily measured in mechanically ventilated patients. By inducing measurable changes in alveolar carbon dioxide concentration, non-shunted pulmonary blood flow to the lungs can be calculated applying a differential Fick principle [10–12].

Our research group has previously described a capnodynamic method that continuously calculates effective pulmonary blood flow (CO_EPBF_) based on small changes in CO_2_ concentration obtained from short expiratory pauses in three out of every nine breaths, conveniently automated by the ventilator [13–15]. CO_EPBF_ can be used as a surrogate for CO when intrapulmonary shunt is low and has shown fair agreement and good trending ability during significant hemodynamic and ventilatory alterations in experimental models [13–16].

Theoretically, the capnodynamic equation is vulnerable to changes in mixed-venous carbon dioxide concentration (CvCO_2_) as the equation system assumes it to remain constant during a measurement cycle. Clinical entities that might cause changes in CvCO_2_ such as reperfusion and hypercapnia are commonly seen in the perioperative period, for example in aortic and laparoscopic surgery. The aim of this study was to evaluate the performance of the capnodynamic method during ischemia–reperfusion and during prolonged hypercapnia induced by an increased dead space in a porcine model.

## Methods

The study was approved by the Uppsala animal research ethical committee (nr. C 47/15, chairperson Kurt Ek) on April 24th 2015 and performed at the Hedenstierna laboratory in Uppsala University, Sweden.

Hällsjö Sander et al. have previously described the anaesthetic and surgical procedure in detail [13]. Briefly, 16 pigs with a mean weight of 36 kg (range 32–44 kg) were anaesthetised and mechanically ventilated in a volume-controlled mode with a tidal volume (TV) of 8 mL/kg, FIO_2_ 0.40 and PEEP 5cmH_2_O (Servo-i, Maquet Critical Care, Solna, Sweden). Core temperature was maintained at 38–39 °C.

Eight animals underwent the ischemia/reperfusion protocol and eight animals the hypercapnia protocol. The animals used in this study were submitted to other separate experimental steps, not affecting the study protocol, that have been published in abstract form [17].

At the end of the protocol animals were sacrificed by a potassium chloride injection.

### Instrumentation and equipment

A 20G catheter was placed in the carotid artery for arterial pressure monitoring and blood gas sampling. A 7.5Fr pulmonary artery catheter (Edwards Lifesciences Corp., Irvine, CA, USA) was inserted via the internal jugular vein for analysis of mixed venous blood gases, pulmonary arterial pressure and cardiac output (CO_PAC_) via thermodilution. A 10Fr, 80 cm thrombectomy catheter (Dispomedica GmbH, Hamburg, Germany) was inserted under ultrasound guidance into the inferior cava vein via the femoral vein to allow controlled preload reduction with balloon inflation.

An ultrasonic flow probe was placed around the pulmonary trunk through a left-sided mini-thoracotomy for CO measurements (CO_TS_) (T 401; Transonic Systems Inc., Ithaca NY, USA).

In the ischemia–reperfusion pigs a 12Fr stent graft balloon catheter (Reliant^®^, Medtronic Inc. Minneapolis, MN, USA) was inserted into the femoral artery with ultrasound guidance and placed in the abdominal aorta just beneath the diaphragm, verified with fluoroscopy.

Expired carbon dioxide was measured by a mainstream infrared sensor (Capnostat-3, Respironics Inc, Wallingford, CT, USA) and gas flow was analysed by the flow sensor incorporated in the ventilator which was connected to a computer where all the mathematical analysis was carried out with a software written in Matlab™ (The Mathworks Inc, Natick, MA, USA).

Blood gas analysis was performed by ABL-800FLEX (Radiometer Medical ApS, Brønshøj, Denmark).

Haemodynamic parameters were retrieved into a data acquisition system (Acknowledge, version 3.2.7, Bio Pac Systems, Santa Barbara, CA, USA).

### Calculations and measurements of cardiac output, shunt and dead space

The capnodynamic method has previously been reported [15] and a detailed description can be found in the supplementary material. Briefly, a short pause is introduced to the expiratory phase of three out of nine breaths, automatically controlled by the ventilator. The resulting small differences (4–8 mmHg) in the alveolar concentration of CO_2_ between breaths can be inserted into the capnodynamic equation, describing the mole balance of CO_2_ transported to and from the lungs. Each breath creates one equation and with a stack of nine equations the CO_EPBF_ can be calculated using a least square-error optimization. With each breath the last equation is replaced with the newest allowing a continuous calculation of CO_EPBF_, the cardiac output minus the intrapulmonary shunt, with each presented value representing an average of nine preceding breaths.

The reference method, CO_TS_, represents the pulmonary blood flow (shunt included) generated by each cardiac cycle measured at the pulmonary trunk. Each measurement was based on an average of approximately 5–10 s.

CO_PAC_, a well-known clinical method to measure CO (shunt included) was used as a reference in case of technical failure. Values were calculated averaging three intermittent thermodilutions always performed after the CO_TS_ and CO_EPBF_ measurements to avoid the short acting effect of cold saline on heart rate.

Shunt fraction was calculated using Berggren’s formula [18].

Enghoff’s dead space was measured using volumetric capnography (NICO monitor, Respironics, Wallingford, CT, USA) [19].

### Experimental protocol

After instrumentation and subsequent 15 min stabilisation, precision and baseline (BL) measurements of CO_TS_, CO_EPBF_ and CO_PAC_ were obtained at PEEP 5cmH_2_O and TV of 8 mL/kg.

### Ischemia/reperfusion

Caudal ischemia was induced in eight pigs by inflation of the aortic balloon for approximately 30 min. The absence of blood flow was confirmed by ultrasound Doppler in the contralateral femoral artery. Severe increases in systemic vascular resistance (SVR) and mean arterial pressure (MAP) during the ischemic period were attenuated with high dose sodium nitroprusside infusion and intermittent beta-blocker boluses. In some animals the aortic balloon was temporarily released and then inflated again if the systolic blood pressure was >200 mmHg, despite maximal pharmacological treatment. Hemodynamic measurements and blood gases were obtained at baseline (BL) and at the end of ischemia, before balloon release. During the reperfusion phase CO_EPBF_ and CO_TS_ measurements were obtained simultaneously every minute and CO_PAC_ at minute 1, 3 and 5.

### Hypercapnia

Prolonged hypercapnia was induced in eight animals by adding an external dead space at the airway opening between the y-piece and the carbon dioxide sensor, aiming for a 50–60% increase in PaCO_2_. Measurements were obtained at baseline, after establishing a stable hypercapnia on average 44 (8) min later and at 7–12 min intervals between baseline, cava balloon inflation, baseline and dobutamine infusion, aiming for ±30% change in CO. CO_EPBF_ and CO_TS_ readings were obtained simultaneously.

### Statistics

D’Agostino and Pearson omnibus K2 test was used to check data for normal distribution. Results are presented as mean (standard deviation, SD). Proportional bias, i.e., the spread of bias at different CO levels, was checked with visual assessment and a linear regression. All statistical calculations except for the polar plots and confidence intervals (CI) were performed in Graph Pad Prism (version 6.0 for Windows, Graph Pad Software, La Jolla, CA, USA). For calculation of polar plots an excel sheet for conversion of Cartesian data to polar coordinates was used (kindly provided by Professor L Critchley) and displayed as graphs in Medcalc Statistical Software version 16.8.4 (MedCalc Software bvba, Ostend, Belgium) [20]. Calculations of CI were performed in excel (version 2007) with a t-table representing degrees of freedom, in accordance with the current discussion on comparison of different cardiac output monitors [21].

The animal was allowed to stabilise during and between each hemodynamic measurement during hypercapnia and therefore a correction for repeated measurements was not applied [21, 22].

### Inherent precision

Inherent precision (defined as twice the coefficient of variation (CV = SD_method_/mean CO_method_) of CO_EPBF_ was calculated from 10 measurements obtained at 1 min intervals in each animal at baseline conditions [23]. The previously reported precision for CO_TS_ was ±10% [24] and between ±8 and ±24% for CO_PAC_ depending on different haemodynamic conditions, ventilation, temperature and positioning of the catheter per se [25, 26]. Precision for CO_EPBF_ was ±14% during steady state conditions in our previous study [15].

### Absolute values

Bland Altman methodology was used to measure the mean difference (bias) and the precision (levels of agreement) between CO_EPBF_ and CO_TS_ [27–29]. Proportional bias was checked with regression analysis to see if the slope deviated from zero. Due to small sample size the mean error (ME), also known as percentage error, used to estimate the precision of agreement was calculated as 100% × t_α,n−1_ × SD_bias_/mean CO_ref_, where t_α,n−1_ is the t-value corresponding to the degrees of freedom (n − 1) and a type I error (α) of 0.05 [21, 29].

Confidence intervals (CI) were calculated for all analyses, as described above. A priori, CO_EPBF_ was considered interchangeable to CO_TS_ if mean error was less than 30% [29], although less than 45% might be considered in settings of extensive hemodynamic changes or apparent advantage of the reference method [30].

### Trending ability

The four-quadrant and the polar plot methodology were used to assess the agreement between test and reference methods regarding the direction and magnitude of the change [20]. Concordance rate for the four-quadrant plot was calculated as the number of data points in the two quadrants of agreement divided by the total number of data points, expressing the agreement between the paired delta-CO values for both methods [20]. Concordance rate for the polar plot was calculated as the number of data points within the radial limits of agreement of ±30° divided by the total number of data points [20].

Because of the high precision of the reference method, <20%, an exclusion zone of 10% was used [31]. We considered a concordance rate of >92 and >90% calculated by the four-quadrant plot and the polar plot methodology as good, respectively [32]. An angular bias smaller than ±5° indicated sufficient calibration between the test and the reference method [20, 32].

## Results

All animals survived the experimental protocol that resulted in notable hemodynamic changes. The calculated inherent precision of CO_EPBF_, CO_TS_ and CO_PAC_ during initial baseline conditions was 8, 4 and 10%, respectively. All data were normally distributed. Proportional bias between CO_EPBF_ and CO_TS_ was undetected in all interventions except during preload reduction in the hypercapnia protocol.

### Ischemia/reperfusion

**Fig. 1:**
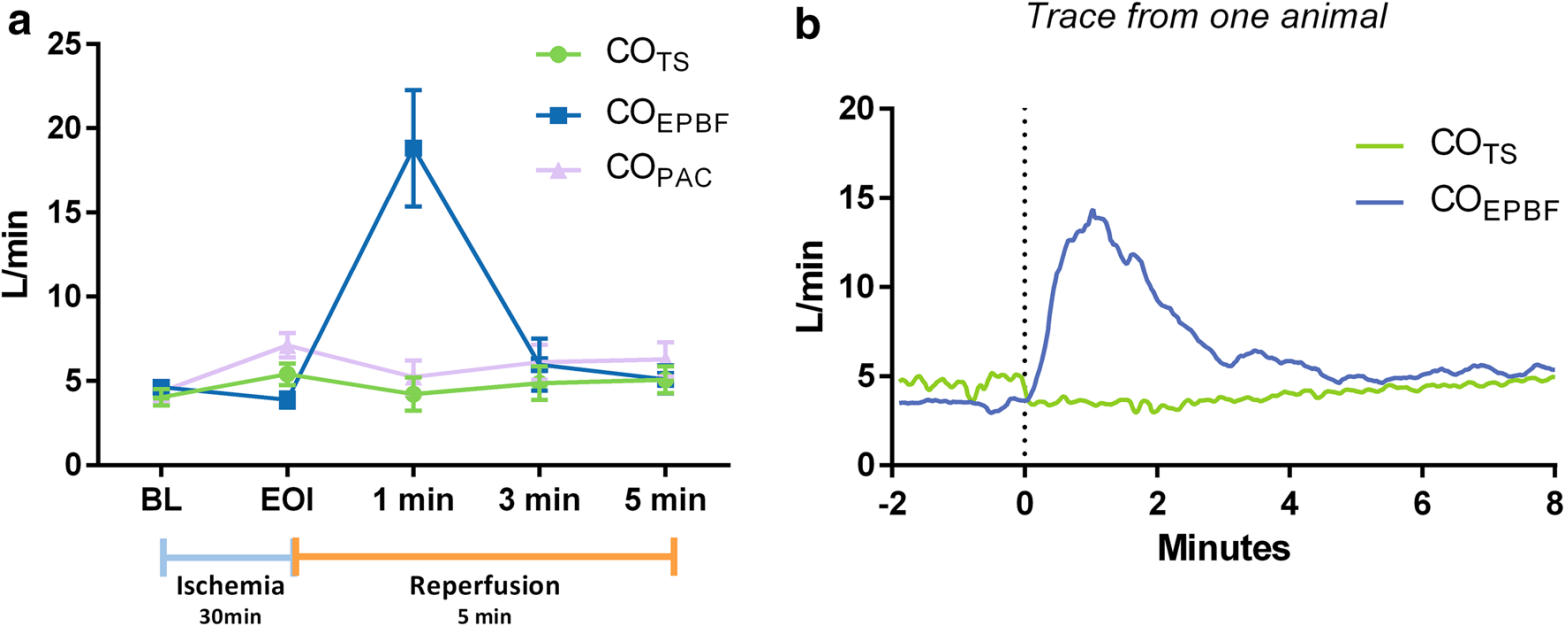
Timeline showing **a** mean (SD) values for CO_EPBF_, CO_TS_ and CO_PAC_ from baseline (BL) to end of ischemia (EOI) approximately 30 min later and at minute 1, 3 and 5 after reperfusion, and **b** continuous values from one animal for CO_EPBF_ and CO_TS_ (not possible with CO_PAC_) from 2 min before balloon release (*vertical broken line*) and up to 8 min after reperfusion

At the end of ischemia, a decrease in PvCO_2_ and increase in serum lactate were observed. Immediately after balloon release, PaCO_2_ and PvCO_2_ increased dramatically and remained high during the initial reperfusion phase (Table 1). Cardiac output varied throughout the protocol (Table 1) and other hemodynamic parameters were largely affected (Table 2). CO_EPBF_ and CO_TS_ showed bias (LoA) 0.7 (−0.4 to 1.8) L/min and ME 28% at baseline. The performance of CO_EPBF_ deteriorated immediately after balloon release but was restored gradually over the next 5 min (Fig. 1; Table 2).

**Table 1:** Cardiac output (L/min) and Bland–Altman values for CO_TS_ and CO_EPBF_ at baseline and during different interventions with confidence intervals (CI) for bias and upper/lower level of agreement (LoA) and mean error (ME)

Condition (n)	CO_EPBF_ (L/min)	CO_TS_ (L/min)	Bias	CI	LoA	CI lower (LoA)	CI upper (LoA)	ME (%)
Baseline (8)	4.5 (0.4)	3.9 (0.5)	0.7	0.3 to 1.1	−0.4 to 1.8	−1.1 to 0.3	1.1 to 2.5	28
End of iscemia (6)	3.9 (0.5)	5.4 (0.6)	−1.5	−2.3 to −0.7	−3.6 to 0.5	−4.9 to −2.2	−0.8 to 1.9	36
Reperfusion 1 min (8)	18.8 (3.4)	4.2 (1.0)	14.6	11.8 to 17.4	6.5 to 22.7	1.7 to 11.3	17.8 to 27.5	186
Reperfusion 3 min (8)	6.0 (1.5)	4.9 (1.0)	1.1	−0.4 to 2.6	−3.3 to 5.5	−5.9 to −0.7	2.9 to 8.1	87
Reperfusion 5 min (8)	5.1 (0.8)	5.1 (0.8)	0.03	−0.7 to 0.8	−2.2 to 2.2	−3.5 to −0.9	0.9 to 3.5	42
Baseline (8)	3.6 (0.5)	3.1 (0.6)	0.6	0.1 to 1.0	0.8 to 1.9	−1.6 to 0.0	1.1 to 2.7	42
Hypercapnia (24)	5.0 (0.7)	4.5 (0.5)	0.5	0.3 to 0.7	−0.5 to 1.4	−0.8 to −0.1	1.1 to 1.7	21
Caval occlusion (8)	3.1 (0.5)	2.7 (0.2)	0.4	0.0 to 0.7	−0.7 to 1.4	−1.3 to −0.1	0.8 to 2.1	38
Dobutamine (8)	7.0 (1.0)	7.04 (0.9)	0.04	−1.0 to 0.9	−2.8 to 2.7	−4.4 to −1.1	1.1 to 4.3	38

**Table 2:** Hemodynamic and metabolic variables during different conditions

	HR (beats/min)	MAP (mmHg)	SVR (dynes/s/cm^5^)	MPAP (mmHg)	Shunt (%)	Dead space (%)	PvCO_2_ (kPa)	PaCO_2_ (kPa)	Lactate (mmol/L)
Baseline	95 (15)	78 (11)	1397 (276)	22 (2)	9 (2)	55 (4)	8.06 (0.69)	6.08 (0.53)	1.5 (0.4)
End of ischemia	133 (8)	115 (28)	1562 (380)	22 (1)	26 (5)	60 (6)	4.35 (2.24)	4.60 (0.33)	8.2 (0.6)
Reperfusion 1 min	117 (12)	48 (8)	758 (131)	21 (3)	19 (6)	67 (5)	10.89 (1.63)	8.4 (1.3)	10.4 (1.2)
Reperfusion 3 min	112 (13)	54 (8)	752 (188)	28 (6)	21 (6)	59 (6)	10.25 (1.61)	8.2 (1.2)	10.4 (1.1)
Reperfusion 5 min	113 (11)	61 (12)	821 (247)	27 (2)	21 (6)	55 (7)	8.75 (1.12)	7.5 (0.92)	10.2 (1.2)
Baseline	75 (9)	65 (7)	1556 (306)	17 (2)	6 (2)	55 (3)	7.43 (0.62)	5.59 (0.42)	2.2 (0.5)
Hypercapnia	97 (12)	78 (9)	1258 (202)	23 (3)	13 (4)	78 (2)	10.50 (0.91)	9.06 (0.49)	0.9 (0.2)
Caval occlusion	98 (16)	49 (5)	1451 (180)	14 (2)	7 (3)	80 (2)	10.33 (0.95)	8.34 (0.42)	0.9 (0.3)
Dobutamine	143 (13)	82 (13)	936 (180)	24 (6)	24 (6)	80 (2)	11.00 (0.44)	9.86 (0.63)	0.8 (0.2)

*HR* heart rate, *MAP* mean arterial pressure, *SVR* systemic vascular resistance, *MPAP* mean arterial pulmonary pressure, *PvCO*_*2*_ partial pressure of CO_2_ in mixed venous blood, *PaCO*_*2*_ partial pressure of CO_2_ in arterial blood

### Hypercapnia

During prolonged hypercapnia, PaCO_2_ and PvCO_2_ increased from 5.6 (0.42) to 9.2 (0.5) kPa, and 7.41 (0.69) to 10.5 (0.91) kPa, respectively. See Table 2 for all hemodynamic, ventilatory and metabolic values.

At hypercapnia baseline conditions, CO_EPBF_ bias (LoA) was 0.5 (−0.5 to 1.4) L/min and ME was 21%.

**Fig. 2:**
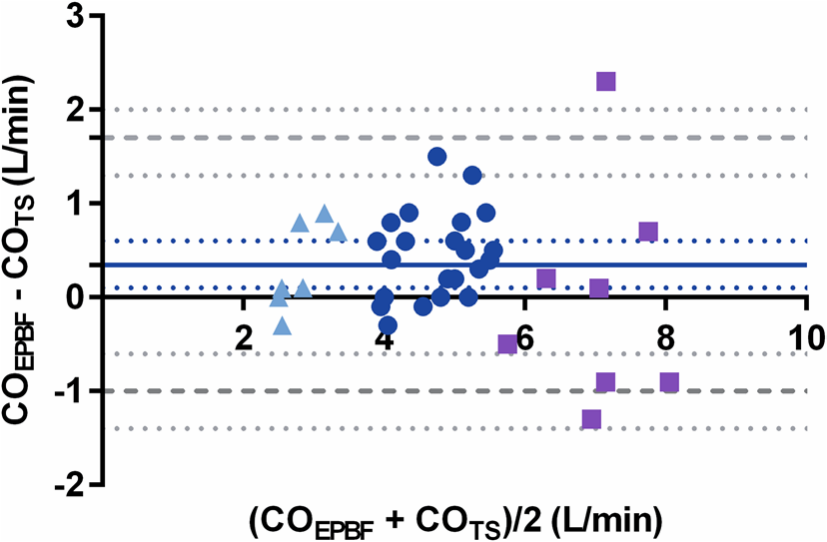
Bland–Altman plot showing 40 paired values for CO_EPBF_ vs CO_TS_ during *dead space induced hypercapnia* at baseline (*blue dots*), low CO (*light blue triangles*) and high CO (*purple quadrants*). Bias is represented with a *whole blue line* with corresponding CI (*blue dotted lines*) and levels of agreement (LoA) are shown with *broken grey lines* with corresponding CI (*grey dotted lines*)

The corresponding values at preload reduction and inotropic stimulation were 0.4 (−0.7 to 1.4) and 0.04 (−2.8 to 2.8) L/min, respectively. ME was 38% in both conditions. See Fig. 2 for visual assessment of the Bland Alman plot during hypercapnia and Table 1 for Bland Altman values for all interventions separately.

### Trending during hypercapnia

**Fig. 3:**
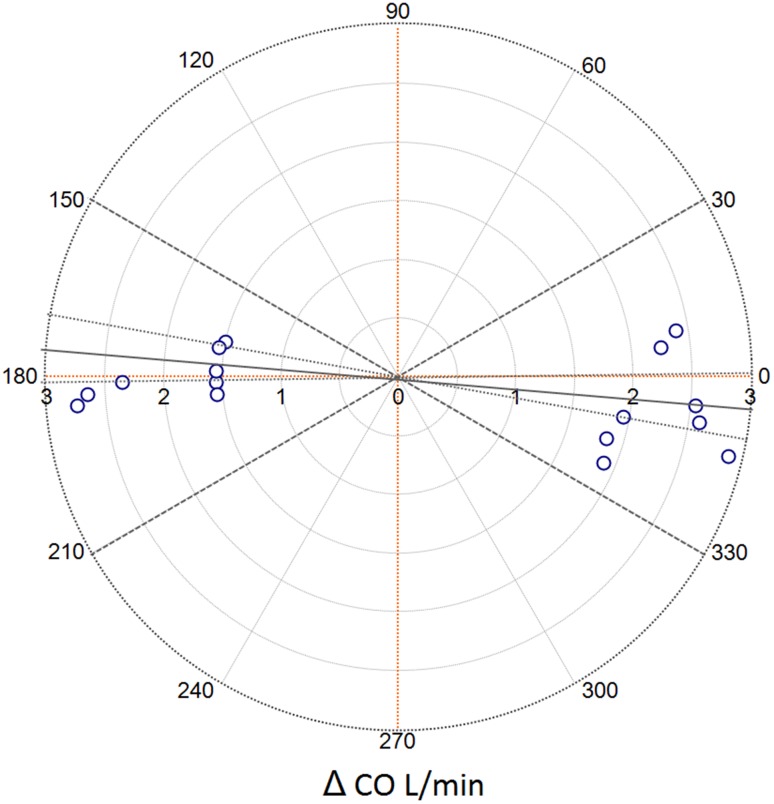
Polar plot for CO_EPBF_ with CO_TS_ as the reference method during *dead space induced hypercapnia*. The radial length is the mean of the pairwise delta values of the reference method and the test method (L/min) and is shown with a *whole black line* with corresponding CI (*dotted lines*). Data spread closely to the polar axis (*orange dotted lines*) indicate good trending

The concordance rate for CO_EPBF_ and CO_TS_ following preload reduction and inotropic stimulation was 100% according to both four quadrant and polar plot methodologies. The mean (95% CI) polar angle was −4.19° (−8.8° to 0.42°) (Fig. 3).

## Discussion

We have evaluated the performance of a capnodynamic method to estimate CO_EPBF_ in a porcine model of ischemia/reperfusion and hypercapnia. As expected, agreement was impaired during reperfusion due to the sudden flux of carbon dioxide into the circulation. However, agreement was restored within 5 min. Prolonged hypercapnia did not impair agreement or trending ability of this method.

Anaesthetists working in the operating room are well aware that rapid physiological changes are to be expected. However, the performance of most non-invasive CO monitors has been found to be less reliable during rapid changes in vascular volume and resistance, a common feature during major surgery [8, 9].

The Fick’s principle, using elimination of CO_2_ from the lungs and the difference in concentration of CO_2_ in mixed venous and arterial blood is a well proven gold standard to calculate CO, although unsuitable for clinical use [33]. Modifications of Fick’s principle based on end tidal CO_2_ measurements, assuming a steady state in C_V_CO_2_, have been used to calculate effective pulmonary blood flow indirectly, which equals CO minus the shunt flow [34]. These indirect methods are dependent on induced alterations in alveolar CO_2_ accomplished by three means; (1) changes in the alveolar ventilation as originally described by Gideon et al. [10] and applied with modifications in our capnodynamic method, (2) changes in dead space as originally described by Capek et al. [35] and used in the NICO system with different software versions [36, 37] and (3) adding carbon dioxide to the inspiratory gas.

The capnodynamic method is continuous with short response time and needs no additional devices mounted to the y-piece, as opposed to the NICO monitor. Originally, inspiratory pauses where used to obtain the desired changes in alveolar ventilation. Good trending was observed during hemodynamic changes and lung injury but agreement (bias) and precision (LoA) was affected at higher PEEP and shunt levels in a porcine model [13, 14]. Following refinement changing to expiratory instead of inspiratory pauses, both agreement and precision improved when compared with the highly invasive ultrasonic flow probe and thermodilution based methods during significant hemodynamic and ventilatory changes [13–16].

A prerequisite for the capnodynamic equation is a stable concentration of C_v_CO_2_ during the measurement cycle. Therefore, we wanted to test the hypothesis that large alterations in venous concentration of carbon dioxide, as might be seen during major surgery, laparoscopy and lung protective ventilation would impair the performance of CO_EPBF_. Rapid influx of carbon dioxide into the circulation is to be expected during vascular surgery following periods of ischemia, whereas in laparoscopic and robotic assisted surgery, insufflation of carbon dioxide induces prolonged hypercapnia. These clinical situations are common features in the operating theatre. The current protocol was designed to evaluate the performance of CO_EPBF_ in an animal model mimicking these clinical scenarios.

In the current study, ischemia and subsequent reperfusion were accomplished by aortic occlusion simulating suprarenal clamping during aortic surgery. The balloon inflation created severe hemodynamic changes in the animal, including a sudden massive increase in systemic vascular resistance demanding treatment with vasodilatory and rate regulatory drugs. As a result, pulmonary shunt increased from 9 to 26% at the end of ischemia and although decreasing during reperfusion, shunt remained elevated from baseline. Since CO_EPBF_ does not detect shunt flow, this contributed to the underestimation of CO at the end of ischemia (see Fig. 1; Table 1). As the capnodynamic equation requires a stable concentration of venous carbon dioxide during the measurement cycle in order to be accurate, CO_EPBF_ increased erroneously following the aortic balloon release due to the resulting dramatic rise in alveolar concentration of carbon dioxide. Despite the massive reperfusion, the performance of CO_EPBF_ was nearly fully restored within 5 min.

Prolonged hypercapnia was induced by adding dead space to the breathing circuit to maintain similar tidal volumes and respiratory rate and to mimic the hypercapnia often seen in laparoscopic surgery. Cardiac output, dead space, shunt, PaCO_2_ and PvCO_2_ increased as expected but did not affect bias, LoA or ME (see Table 2). These results indicate that the concentration of carbon dioxide per se does not impair the performance of the method in contrast to sudden changes. The performance of many CO monitoring techniques is impaired during rapid hemodynamic changes and requires frequent calibration in order to maintain accuracy. The capnodynamic method recuperated within 5 min after reperfusion. This time span is comparable to the duration of the calibration procedures of the thermodilution based CO monitors [30, 38].

The capnodynamic method also includes an internal control function that detects major incongruities that do not fit with the ideal capnodynamic equation. The inherent control function, not used in this study, has the potential to filter out unstable values as those seen in the reperfusion phase, supporting the clinician with only stable calculations of pulmonary blood flow.

In the statistical analysis we adapted to the most recent guidelines on comparing different cardiac output monitors [21]. Taken our previous studies and animal-research ethics into account we used as few animals as possible, despite an expected effect on confidence intervals and higher risk for proportional bias. The physiological stresses imposed on the animals in this study were extreme and the results needs to be interpreted in this context. At baseline the capnodynamic method showed acceptable bias, LoA and ME. Five minutes after massive reperfusion, notable increase in CO and large reduction in SVR the bias was almost zero but LoA and ME had increased. We believe this is acceptable for a continuous minimally invasive method complimentary to mechanical ventilation in anesthetized subjects, as long as trending is preserved, as previously suggested by Peyton and Chong [30].

This study has several limitations. As the capnodynamic method measures carbon dioxide in expired gas, the mixed-venous carbon dioxide bypassing the lungs i.e., shunted, is not detected. In the current study, we have utilized CO as a reference for comparison. Since the reference method and the test method do not measure identical physiological variables, a difference is to be expected. Our previous studies have shown that CO_EPBF_ has acceptable agreement when shunt is below 20%. At higher shunt fractions the agreement is impaired, however, the trending ability is still reliable [13–15].

The major hemodynamic changes in the protocol required intermittent intense pharmacologic support. This resulted in inter-individual differences in treatment and physiological status between animals and thus, a less homogenous model.

It could also be reasoned that with the magnitude of these circulatory changes, the trending ability is anticipated to be aligned. However, we believe that it is of utmost importance to challenge new monitoring technologies in extreme conditions which is ethically not possible to induce in humans.

In conclusion CO_EPBF_ maintained a good agreement during hypercapnia and was marginally affected during severe ischemia. Only 5 min after massive reperfusion agreement returned to previous acceptable levels. Clinical studies on CO_EPBF_ are underway.

## Electronic supplementary material

Below is the link to the electronic supplementary material.

Supplementary material 1 (DOCX 15 KB)
