# Supplementary material for: Performance of a capnodynamic method estimating effective pulmonary blood flow during transient and sustained hypercapnia
Source: J Clin Monit Comput. 2017 May 11;32(2):311–9. doi: 10.1007/s10877-017-0021-3 (PMC5838142; doi:10.1007/s10877-017-0021-3)
Supplement: Supplementary file 1 — Supplementary material 1 (DOCX 15 KB) [file 10877_2017_21_MOESM1_ESM.docx]

**Supplementary material**

**Calculation of effective pulmonary blood flow**

The capnodynamic equation (see below) describes a mole balance of carbon dioxide between the transport of carbon dioxide to and from the lung and the rate of change of the carbon dioxide content in the lung achieved by a superimposed breathing pattern. The pattern consists of total nine breaths; six normal breaths and three breaths with a 3-4 seconds expiratory pause. This creates a difference in mean alveolar ventilation (ca 4-8 mmHg) which can be inserted into the capnodynamic equation on the left side of the equal sign along with effective lung volume (ELV) thought to represent the gas volume in the lung containing CO_2_. On the right of the equal sign, EPBF represents the circulatory supply of CO_2_ to the lung, Δt^n^ expresses the duration of each breathing cycle, which varies due to the imposed breath holds and C_v_CO_2_ represents the amount of CO_2_ in mixed venous blood and is assumed to be constant during a measurement cycle.

The carbon dioxide content of pulmonary end-capillary blood, CcCO_2_, is derived from the measured alveolar partial pressure (P_A_CO_2_) using a dissociation curve for CO_2_ in blood adapted from Capek et al with regards to measured haemoglobin obtained in the beginning of the experiment.^33^ The term VTCO_2_^n^ displays the quantity of carbon dioxide exhaled by the n^th^ tidal volume. EPBF, ELV and are all calculated from the equation system using a least square-error optimisation of the fit between the left and right side of the equation.

$$ELV\cdot\left(F_{A}CO_{2}^{n}-F_{A}CO_{2}^{n-1}\right)=EPBF\cdot\Delta t^{n}\cdot\left(C_{v}CO_{2}-C_{c}CO_{2}^{n}\right)-VTCO_{2}^{n}$$

ELV Effective lung volume [L]

EPBF Effective pulmonary blood flow [L/min]

n current breath

n-1 previous breath

F_A_CO_2_ mean alveolar carbon dioxide fraction

C_v_CO_2_ mixed venous carbon dioxide content [L_gas_/L_blood_]

C_c_CO_2_^n^ pulmonary end-capillary carbon dioxide content [L_gas_/L_blood_]

VTCO_2_^n^ volume [L] of carbon dioxide eliminated by the current, n^th^, breath

Δt ^n^ current breath cycle time [min]

A prerequisite for the equation is the assumption that ELV, EPBF and C_v_CO_2_ remain constant during the complete measurement cycle consisting of a set of nine breaths. The method is continuous because the measurement cycle is constantly updated with the most recent equation expelling the last. A correction for the shunt fraction is not included.
